# Transcatheter Tricuspid Valve Replacement: Current Evidence, Device Landscape, and Future Directions

**DOI:** 10.7759/cureus.101389

**Published:** 2026-01-12

**Authors:** Ebrahim K Al-Ebrahim, Tahir Mohamed

**Affiliations:** 1 Internal Medicine, King Faisal Specialist Hospital and Research Centre, Riyadh, SAU; 2 Cardiology, King Faisal Specialist Hospital and Research Centre, Riyadh, SAU

**Keywords:** tanscatheter, tricuspid valve, tricuspid valve repair, ttvr, valve replacement

## Abstract

Transcatheter tricuspid valve interventions have rapidly evolved over the last decade in response to the large, historically undertreated population with severe tricuspid regurgitation (TR). While most early experience focused on leaflet approximation and annuloplasty, orthotopic transcatheter tricuspid valve replacement (TTVR) has emerged as a transformative strategy that can achieve near-complete elimination of TR in anatomies that are suboptimal for repair. This narrative review summarizes the epidemiology and pathophysiology of severe TR, the rationale for valve replacement, contemporary TTVR devices and implantation concepts, available clinical evidence, and remaining challenges and future directions.

## Introduction and background

Tricuspid regurgitation (TR) is common, especially in elderly patients with left-sided valvular disease, left-sided heart failure or pulmonary hypertension, or device leads. Severe TR is associated with progressive right-sided heart failure, liver and renal dysfunction, impaired functional capacity, and excess mortality [1]. Perioperative mortality with isolated tricuspid surgery remains substantial in high-risk populations, which has driven intense interest in transcatheter tricuspid valve interventions [2]. Most transcatheter experience to date has been with repair technologies, such as edge-to-edge repair and annuloplasty, which are particularly suitable for patients with moderate annular dilatation and smaller coaptation gaps [3]. However, many patients present with massive or torrential TR, very large annuli, or complex tethering in whom repair alone is unlikely to restore competence. In this context, orthotopic transcatheter tricuspid valve replacement (TTVR) has emerged as an attractive alternative, offering the concept of “surgical-type” TR elimination without sternotomy and cardiopulmonary bypass. The first-in-human experiences, small registries, and more recently randomized trial data have progressively defined the role of replacement in the broader tricuspid toolbox [3].

## Review

Pathophysiology of functional TR and rationale for replacement

Most clinically significant TR is functional, arising from annular dilatation and leaflet malcoaptation due to right atrial enlargement, RV remodeling, or both [4]. Progressive volume overload leads to further chamber dilation, leaflet tethering, and worsening regurgitation, establishing a vicious cycle. In advanced stages, massive and torrential TR result in extreme right-sided volume overload, systemic venous congestion, and multiorgan dysfunction. Leaflet tissue is often thin and fragile, and the annulus is non-circular and highly dynamic, particularly in the septolateral dimension [4].

Transcatheter repair addresses TR either by reducing the effective regurgitant orifice (leaflet approximation) or annular size, but depends on adequate leaflet tissue, limited tethering, and manageable coaptation gaps ≤ 7mm [5]. In contrast, TTVR aims to replace the native valve with a competent bioprosthesis that decouples competence from native leaflet geometry, potentially offering more predictable TR elimination. From an anatomical perspective, annular size and geometry are key determinants of suitability for TTVR [5]. A dilated annulus within the range for secure anchoring favors procedural success, whereas extreme enlargement increases the risk of malposition. To expand eligibility, newer systems such as EVOQUE (Edwards Lifesciences, Irvine, CA, USA) provide larger implant options, including a 56-mm valve, for selected patients with large annular anatomies [5].

Nevertheless, sudden elimination of severe TR also abruptly increases RV afterload, and careful assessment of RV function and pulmonary pressures is essential to avoid precipitating RV failure [5].

Device concepts and procedural principles

Imaging guidance is crucial. Pre-procedural multidetector computed tomography (CT) defines annular dimensions, RV geometry, relation to the right coronary artery, and interaction with pacing leads. Intraprocedurally, transesophageal echocardiography and fluoroscopy guide coaxial alignment, depth, and deployment. Device selection is individualized based on anatomy, annular size, RV configuration, and presence of trans-tricuspid leads (Table 1).

**Table 1:** Device Concepts and Procedural Principles of Transcatheter Tricuspid Valve Replacement Systems

Device	Access Route	Key Procedural / Design Features
EVOQUE [5]	Transfemoral, transjugular, or trans-axillary venous	Large-bore steerable delivery sheath; atrial-then-ventricular deployment sequence
NaviGate / GATE [6]	Trans-atrial or transjugular venous	Self-expanding nitinol frame with equine pericardial leaflets
LuX-Valve [7]	Trans-atrial (right atrial); newer iterations via transjugular	Annular anchoring with dedicated septal anchor for prosthesis stabilization
LuX-Valve Plus [7]	Trans-atrial or transjugular	Similar anchoring concept with refinements for transjugular delivery

EVOQUE Transcatheter Tricuspid Valve Replacement System

The EVOQUE valve is a self-expanding nitinol frame with bovine pericardial leaflets and an atrial skirt designed to conform to the large, non-calcified tricuspid annulus. It was the first TTVR system to receive regulatory approval in multiple regions, following a structured program of early feasibility and pivotal trials [8].

Initial single-arm experiences demonstrated high procedural success, marked reduction of TR to mild or less in the vast majority of patients, and early symptomatic improvement [9]. Subsequently, the pivotal randomized TRISCEND II trial compared EVOQUE plus optimal medical therapy with medical therapy alone in patients with severe or greater symptomatic TR at prohibitive surgical risk. At one year, treatment with EVOQUE resulted in meaningful improvements in quality of life and functional endpoints, mainly attributable to increases in Kansas City Cardiomyopathy Questionnaire scores and functional capacity, without significant differences in mortality compared with controls [10]. TR reduction was dramatic and durable, with the vast majority of patients having residual TR graded as mild or less after one year [10].

LuX-Valve and LuX-Valve Plus

The LuX-Valve (Jenscare Scientific, Suzhou, China) family represents another major TTVR platform. The original LuX-Valve is a self-expanding bioprosthesis with an atrial disc, a ventricular anchor that grasps the septal leaflet and septum, and a unique design aimed at stabilizing the prosthesis in the large tricuspid annulus without relying on radial force alone. Early clinical series in high-risk patients with severe or torrential TR showed high procedural success [11].

LuX-Valve Plus is an evolution of the platform with modifications to facilitate fully transjugular delivery and accommodate very large right-sided anatomies. Early data from dedicated trials and registries, including cohorts with torrential TR and complex anatomies, indicate high implantation success, substantial TR reduction, and favorable early clinical event rates. Short-term follow-up suggests early reverse remodeling of the right atrium and right ventricle and improvement in symptoms, but longer-term durability and late complications such as structural valve deterioration or thrombosis remain to be defined [11,12]. Ongoing studies will help clarify how LuX-Valve-based therapies compare with other TTVR systems and with advanced repair strategies in different clinical and anatomical subsets.

NaviGate/GATE and Other Orthotopic Valves

The NaviGate or GATE (NaviGate Cardiac Structures, Lake Forest, CA, USA) tricuspid valved stent is among the earlier orthotopic TTVR devices. It is a self-expanding nitinol frame with tri-leaflet equine pericardial tissue, designed to anchor at or slightly below the tricuspid annulus using a combination of atrial and ventricular components. Initial first-in-human experience in patients with severe functional TR at high surgical risk demonstrated technical feasibility, meaningful TR reduction, and early symptomatic benefit [8].

Patient selection and imaging for TTVR

Careful patient selection is central to successful TTVR. Candidates are typically patients with symptomatic severe, massive, or torrential TR who remain limited despite guideline-directed medical therapy and are at high or prohibitive risk for surgery [1]. Important considerations include:

Right ventricular function: Severe RV dysfunction may not tolerate the abrupt increase in afterload that follows TR elimination. Multimodality imaging, including echocardiographic strain and cardiac magnetic resonance when feasible, helps assess RV reserve.

Pulmonary vascular disease: Very high pulmonary vascular resistance raises concern for RV failure after TTVR [1,5].

Anatomic suitability: CT and echocardiography define annular size, leaflet tethering, coaptation gap, RV geometry, and relation to the right coronary artery. Thresholds for annular dimensions and coaptation length vary by device [1,5].

Presence of trans-tricuspid leads: Options include jailing the lead between the stent frame and annulus, extracting the lead before TTVR, or combining with leadless pacing.

End-organ function: Hepatic and renal impairment are common in advanced TR and influence risk-benefit assessment [1,5].

Multimodality imaging is used not only for screening but also for procedural planning and intraprocedural guidance. Dedicated CT protocols reconstruct the entire right heart, assess landing zone relationships, and simulate device fit. During the procedure, transesophageal or intracardiac echocardiography combined with fluoroscopy guides steering, coaxiality, depth, and confirmation of TR reduction and valve function [5]. The implementation of three-dimensional intracardiac echocardiography (ICE) catheters has substantially increased procedural success during TTVI, especially in patients with limited or poor-quality guidance from transesophageal echocardiography [13].

Clinical outcomes and complications

Across devices and studies, TTVR has consistently achieved high procedural success and near-complete TR elimination in the majority of treated patients [14,15]. In contemporary series and randomized trials, most patients experience substantial improvements in NYHA functional class, six-minute walk distance, and health status measures within months of the procedure [16]. Early evidence also suggests favorable reverse remodeling of the right atrium, right ventricle and improvement of markers of hepatic congestion in many patients [17].

However, TTVR is a complex intervention with a distinct complication profile. Major vascular and bleeding complications remain a concern due to large-bore venous access and atrial manipulation [18]. New conduction abnormalities and need for permanent pacemaker implantation are not uncommon, especially with devices that extend toward the septal region or interact closely with the His bundle and right bundle branch. Device malposition, embolization, and paravalvular leak are rare but serious events that require immediate recognition and management [19].

TTVR versus transcatheter repair

Transcatheter repair and replacement should be seen as complementary modalities rather than competitors. Edge-to-edge repair and annuloplasty have demonstrated safety and symptomatic benefit in selected patients, particularly earlier in the disease course with moderate annular dilatation, smaller coaptation gaps, and preserved leaflet mobility [20]. These procedures typically carry lower device profile, less need for large-bore access, and relatively low rates of conduction disturbance [20].

Conversely, TTVR offers more predictable elimination of TR and is often favored in patients with advanced disease, massive or torrential TR, extreme annular dilation, and complex leaflet tethering where repair is unlikely to restore competence [21].

Current guidelines and consensus documents emphasize the importance of heart-team decision-making, integrating clinical status, anatomy, RV function, comorbidities, and patient preferences in selecting between repair and replacement [22].

Future comparative studies and randomized trials will be crucial to refine these treatment algorithms. Ultimately, a personalized, anatomy- and physiology-guided strategy is likely to prevail, with repair, replacement, or combinations thereof deployed at different stages of the disease trajectory.

Future directions

Several key questions remain open in the field of TTVR. First, the long-term durability of right-sided bioprostheses under low-pressure but high-volume conditions is unknown; extended follow-up will be needed to understand structural valve degeneration patterns, optimal antithrombotic strategies, and the feasibility of future valve-in-valve procedures. Second, better tools for assessing RV function and reserve are needed to optimize patient selection and timing of intervention, potentially avoiding late presentation when irreversible RV failure has already developed [22].

Fourth, device designs will continue to evolve toward lower-profile delivery systems, more flexible frames to accommodate extreme anatomies, and dedicated solutions for patients with pacemaker or defibrillator leads traversing the tricuspid valve. Finally, health-economic analyses and real-world registries will be essential to understand cost-effectiveness, access, and equity in the dissemination of these complex therapies.

From a research perspective, ongoing and planned randomized trials will help clarify the relative merits of TTVR versus medical therapy and versus repair, as well as define optimal endpoints that capture the multi-organ benefits of TR correction [21,22]. Furthermore, deeper mechanistic studies of RV adaptation, venous congestion, and end-organ recovery after TTVR may reveal biomarkers and imaging signatures that guide timing and intensity of intervention.

## Conclusions

Transcatheter tricuspid valve replacement has moved rapidly from early feasibility into a maturing therapeutic option for patients with severe, symptomatic TR at high surgical risk, particularly those with anatomies not suitable for repair. Contemporary devices such as EVOQUE, LuX-Valve, NaviGate/GATE, and other orthotopic platforms achieve high procedural success and consistent TR elimination, translating into substantial improvements in symptoms, functional capacity, and quality of life in the short- and mid-term. At the same time, TTVR carries a non-negligible risk of bleeding, conduction disturbances, and potential RV failure in vulnerable patients, underscoring the need for careful patient selection and expert heart-team evaluation.

As evidence accumulates from randomized trials, large registries, and longer-term follow-up, TTVR is likely to become an integral component of a comprehensive, transcatheter-based strategy for the management of tricuspid valve disease.
